# Adipose Stem Cells in Regenerative Medicine: Looking Forward

**DOI:** 10.3389/fbioe.2021.837464

**Published:** 2022-01-13

**Authors:** Sara Al-Ghadban, Maria Artiles, Bruce A. Bunnell

**Affiliations:** Department of Microbiology Immunology and Genetics, University of North Texas Health Science Center, Fort Worth, TX, United States

**Keywords:** adipose-derived stem cells (ASCs), regenerative medicine, tissue engineering, scaffolds, microfluidic systems

## Abstract

Over the last decade, stem cell-based regenerative medicine has progressed to clinical testing and therapeutic applications. The applications range from infusions of autologous and allogeneic stem cells to stem cell-derived products. Adult stem cells from adipose tissue (ASCs) show significant promise in treating autoimmune and neurodegenerative diseases, vascular and metabolic diseases, bone and cartilage regeneration and wound defects. The regenerative capabilities of ASCs *in vivo* are primarily orchestrated by their secretome of paracrine factors and cell-matrix interactions. More recent developments are focused on creating more complex structures such as 3D organoids, tissue elements and eventually fully functional tissues and organs to replace or repair diseased or damaged tissues. The current and future applications for ASCs in regenerative medicine are discussed here.

## Introduction

ASCs and ASC-derived extracellular vesicles (ASC-EVs) and ASC conditioned media (ASCs-CM) have been extensively studied and widely used in regenerative medicine. Studies have shown the effectiveness of ASCs and ASCs secretome (Zuk et al., 2001; Giannasi et al., 2020; Trzyna and Banaś-Ząbczyk, 2021) therapy in numerous diseases such as cardiovascular, bone regeneration, osteoarthritis graft versus host disease (GvHD) and autoimmune disorders such Crohn’s diseases (Galindo et al., 2017), systemic lupus erythematosus (SLE) and multiple sclerosis (Maria et al., 2017; Al-Ghadban and Bunnell, 2020). In addition, researchers have recently been designing new 3D biomaterials by combining ASCs with biomimetic scaffolds composed of either natural or synthetic materials. These 3D biomaterials have proven effective in tissue repair and organ regeneration (Storti et al., 2019; Gibler et al., 2021). These scaffolds have biological and physical properties that imitate the native ECM niche, which is crucial for stem cell adhesion, growth, proliferation and differentiation along particular lineages. Researchers are also incorporating ASCs into novel microfluidic systems, also known as organ-on-a-chip models, to model diseases in systems that function as intact organs, as substitutes for living organisms for testing of new therapeutic interventions (Zhang et al., 2017; Mofazzal Jahromi et al., 2019; O’Donnell et al., 2020). The content of this review is focused on the current and future applications of ASCs in regenerative medicine. The review also presents strategies and challenges in this field and explores the potential advancement of tissue engineering for clinical applications.

## ASC Properties

Adipose tissue is distributed throughout the body in tissues including the bone marrow, beneath the skin (subcutaneous), within joints (intra-articular) and around internal organs (visceral adipose tissue). Adipose tissue is also located in ectopic sites, including the liver (intra-hepatic) and muscle (intra-muscular). Adipose tissue was viewed as an inactive organ that functioned primarily as an energy reservoir for the longest time (Trayhurn and Wood, 2005; Cawthorn et al., 2012). However, the initial identification of leptin, a cytokine produced by adipose tissue, and subsequently numerous other adipokines, led to the re-classification of adipose tissue as an endocrine organ (Zhang et al., 1994). Adipose tissue can also produce several other cytokines, including pro-inflammatory mediators such as IL-6, TNF-a, IL-1b, IL-8, and MCP-1, that drive inflammation (Coppack, 2001; Caer et al., 2017).

**GRAPHICAL ABSTRACT:**
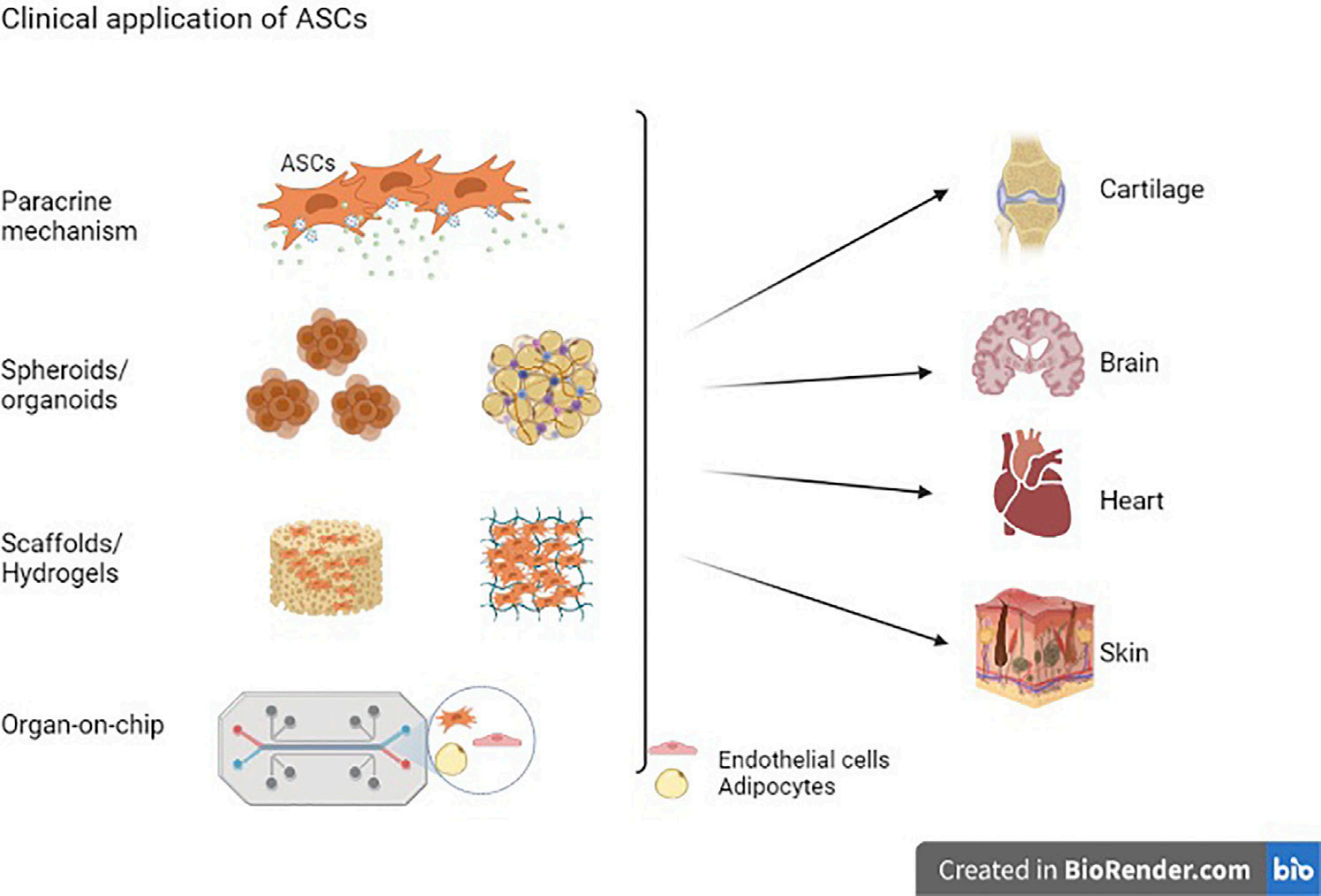


Three distinct types of adipose tissue have been described in humans. White adipose tissue (WAT), localized subcutaneously or in depots within the abdomen, comprises adipocytes and functions as an energy storage depot. Brown adipose tissue (BAT) is distributed throughout the body in interscapular regions, supraclavicular, suprarenal, pericardial, para-aortic and around the pancreas, kidney and trachea. BAT has thermogenic activity induced by shivering and non-shivering mechanisms. The thermogenic activity of BAT is driven by the expression of the mitochondrial membrane protein Uncoupling Protein 1 (UCP1).

In comparison, beige (“brite” or “brown/white”) adipose tissue typically localizes with WAT. It serves as an energy storage depot that can express UCP1 and have thermogenic activity. The most common source of adipose tissue used to isolate ASCs is subcutaneous WAT collected from the abdomen, thigh, hips, or buttocks, typically during plastic surgical procedures.

Mature adipocytes are the primary cellular component of adipose tissue; however, adipose tissue represents a heterogeneous population of cells. Adipose tissue is comprised of preadipocytes, pericytes, fibroblasts, smooth muscle cells, endothelial cells, hematopoietic cells, mature immune cells such as B and T-cells, macrophages, myeloid cells and adipose tissue-derived mesenchymal stem cells (ASC).

ASCs are fibroblast-like cells isolated from the stromal vascular fraction (SVF) isolated by processing the adipose tissue (AT) by either enzymatic or mechanical methods. ASCs, comprise roughly 1–10% of the SVF. ASCs adhere to tissue culture plastic, proliferate in cell culture, undergo self-renewal, and effectively differentiate into multiple lineages, *in vitro* and *in vivo.* ASCs have been reported to differentiate into adipocytes, chondrocytes, osteoblasts, cardiomyocytes, skeletal muscle cells, neurons, hepatocytes and tenocytes, at least *in vitro* (Aurich et al., 2009; Vallée et al., 2009; Choi et al., 2010; Harris et al., 2011; Gimble et al., 2017; George et al., 2018; Mohiuddin O. A. et al., 2019; Lee et al., 2020; Harrison et al., 2022).

Flow cytometric analysis of the cell surface cluster of differentiation (CD) antigens is used to characterize ASCs. The majority of CD antigens screened are canonical markers of mesenchymal lineage cells. To date, an ASC-specific CD marker has not been identified that explicitly identifies ASCs. The analysis typically includes screening for both positive and negative CD antigens. Human ASCs are positive for known mesenchymal stem cell surface markers, including the cell adhesion molecules CD29, CD44, CD146, and CD166; the receptor molecules CD90 and CD105; and the GPI anchored enzyme CD73. In addition, ASC should be negative (< 2%) for the hematopoietic cell surface antigens, including CD11b, CD13, CD14, CD19, and CD45. They do not express the endothelial markers (CD31) and the human leukocyte antigen (HLA)-DR (Tucker and Bunnell, 2011; Al-Ghadban and Bunnell, 2020; Harrison et al., 2022). ASCs are unique in their expression of CD36 (fatty acid translocase) and the absence of vascular cell adhesion molecule (VCAM-1/CD106) (Rajashekhar et al., 2008; Baer et al., 2013). It is worth noting that ASCs isolated from two different subcutaneous adipose tissue depots (abdomen and thigh) express the same CD markers and demonstrated a similar multi-differentiation potential (Choudhery et al., 2015). However, ASCs isolated from subcutaneous adipose tissue showed functional differences such as gene expression, growth factor secretion, proliferation rates and differentiation potential compared to ASCs isolated from visceral AT, suggesting that ASCs isolated from different depots may have unique properties (Hocking et al., 2010; Baglioni et al., 2012; Ong et al., 2014). Despite the differences in their characteristics, ASCs isolated from different depots have been used successfully in both clinical research and tissue engineering applications.

## Application of ASCs for Regenerative Medicine

Over the last decade, ASCs have been broadly applied in regenerative medicine applications. ASCs secrete numerous soluble mediators, inflammatory cytokines, angiogenic, trophic and growth factors such as vascular endothelial growth factor (VEGF), hepatocyte growth factor (HGF), brain-derived neurotrophic factor (BDNF), insulin-like growth factor (IGF), basic fibroblast growth factor (bFGF), transforming growth factor beta 1 (TGF-β1), stromal cell-derived factor (SDF)-1α, and interleukins. The paracrine factors secreted by ASCs contribute to tissue repair, wound healing and organ regeneration (Kapur and Katz, 2013; Suga et al., 2014; Brini et al., 2017; Li et al., 2019; Trzyna and Banaś-Ząbczyk, 2021). Studies have also shown that ASCs-EVs stimulate the regeneration of damaged tissue similar to the ASCs (Hu et al., 2016; Qiu et al., 2018). Basalova et al., have shown that ASCs-EV reduced fibrosis by stimulating myofibroblast dedifferentiation (Basalova et al., 2020). Other studies have also demonstrated the immunomodulatory effect of ASCs conditioned media in tissue repair and therapy (Yuan et al., 2018). In addition to the 2D monolayer cell cultures and 3D scaffolds extensively used to study the ASCs, an “organ-on-a-chip” technology is emerging as an advanced technique in stem cell-based therapy and will be discussed in this paper.

## Current Therapeutic Applications of ASCs

### Fat Grafting and Tissue Reconstruction

Fat grafting is a procedure that was initially attempted in 1889. Since then, many attempts have been made to create a standard protocol that will provide ideal outcomes. Cell-assisted lipotransfer (CAL), SVF and ASCs have demonstrated promise in adipose tissue regeneration and augmentation (Zhu et al., 2015; Li et al., 2017; Hong, 2020). The administration of ASCs enhances the outcomes of fat grafting in humans, particularly breast reconstruction, facial reconstruction and cosmetic surgery applications (Shukla et al., 2020b; Fang et al., 2021). This is mainly due to the ability of ASCs to differentiate into endothelial and epithelial cells as well as secrete cytokines and growth factors that promote angiogenesis through paracrine mechanisms and cell-cell interactions in a co-culture system, thus enhancing neovascularization and accelerating wound healing (Nie et al., 2011; Haubner et al., 2013). A study conducted by Kim et al. showed that ASCs intravenously injected in mice migrate to the wound area and promote tissue repair by differentiating into epithelial cells, thus inducing cutaneous regeneration by re-epithelialization of the dermal layer (Kim et al., 2019). Another study by Yu et al. and others demonstrated that transplanted ASC sheets enhance wound healing and reduce scar formation in a nude mice model. In this study, ASCs decreased inflammation and increased cellular viability in damaged tissue (Yu et al., 2018). In addition, fat grafting has shown to be an alternative for patients with wounds that do not respond to traditional therapy. A case report conducted by Vyas et al. demonstrated the ability of ASCs to promote wound healing in a patient suffering from a radiation-induced wound (Vyas et al., 2021). Furthermore, the use of lipoaspirate transplants containing ASCs has been shown to reverse skin necrosis after irradiation-induced degenerative chronic lesions in tissues exposed to oncologic radiotherapy (Rigotti et al., 2007).

Currently, cosmetic surgery represents the highest demand for ASCs, primarily for autologous transfers, with breast reconstruction and facial rejuvenation being the most popular applications (Bora and Majumdar, 2017). Fat reconstruction is typically used in facial soft-tissue repairs and to rebuild tissues subjected to surgical oncological treatments (Li et al., 2017). Nevertheless, the success rate of fat transplants varies between patients, which might be due to several factors, including the methods of ASCs isolation, surgical procedure, and the site of fat transplantation. One of the challenges of autologous fat grafts is fat reabsorption, decreasing the total volume of transplanted fat grafts by 20–70% (Bellini, 2017). However, the use of platelet-rich plasma (PRP), b-FGF, VEGF and estradiol as supplements for ASCs culture has demonstrated the ability to decrease the rate of fat reabsorption and enhanced fat transplant (Yuksel et al., 2000; Pires Fraga et al., 2010; Luo et al., 2013; Li et al., 2017).

### Cardiovascular Diseases

ASCs have been demonstrated to mediate improvements after myocardial infarction via their inherent anti-apoptotic, anti-inflammatory, and pro-angiogenic effects. ASCs also contribute to inhibiting fibrosis and cardiac remodeling through the recruitment of endogenous stem cells and influence their re-entry into the cardiovascular lineage cell cycle (Li et al., 2019a). In a rat model of chronic ischemic cardiomyopathy (ICM), a group of researchers sought to identify early changes in cardiac cellular subpopulations and transcription after treatments with a well-characterized and pure cryopreserved allogeneic ASCs isolated from male Lewis (Follin et al., 2021). Treatment with ASCs resulted in altered inflammatory monocyte/macrophage subpopulations, increased CD4/CD8 ratio, and an increased uncharacterized CD31−CD45−CD90− population. Also, colony-stimulating factor 2, VEGFA and VEGFB were upregulated in the treated animals. These changes are associated with positive chemotaxis, monocyte and macrophage differentiation, and angiogenesis. These results indicate that some of the immediate effects of ASCs were related primarily to monocyte/macrophage regulation (Follin et al., 2021). The first human clinical trial report on the use of cryopreserved off-the-shelf ASCs in ischemic heart disease and heart failure treatment was published in 2017 (Kastrup et al., 2017). The study included ten patients ranging in ages from 30 to 80 years old. The follow-up was done at 1, 2, 3, and 6 months. ASCs were obtained from lipoaspirates from three healthy female donors of ages between 28 and 33 years old. After the 6-months follow-up, overall cardiac function showed a tendency to improve, with left ventricle pump function and a reduction in dilatation of the left ventricle. Interestingly enough, while two patients had donor-specific HLA antibodies at baseline and four patients developed donor-specific *de novo*, this did not affect the efficacy of the treatment, nor were there any significant adverse events post-treatment, suggesting that even with allogeneic ASCs transplantation, immunosuppressant drugs may not be required.

### Bone Defects

Bone tissue has an inherent ability to repair and regenerate; however, the “critical-size bone defect” concept limits the extent of this regeneration potential. This concept simply refers to the point at which the extension of the lesion is too great for the effective signal transduction and delivery of growth factors to drive the regeneration. At this point, scaffolds combined with stem cells such as ASCs are required (Mohiuddin O. A. et al., 2019; Storti et al., 2019). Nonetheless, bone-derived grafts are the second most transplanted material, next to blood. Only 28% of patients with open fractures can make a full recovery (Alonso-Goulart et al., 2021) on their own. Bone tissue is composed of three types of cells that maintain its integrity: osteoblast, osteoclast, and osteocyte cells. Bone remodeling and regeneration require the interaction between osteoclasts and osteoblasts (Baddour et al., 2012). Several studies have shown that ASCs can be differentiated into osteoblasts. Adding bone morphogenetic protein 2 (BMP-2), growth factors, PRP, extracellular calcium enhances their differentiation and bone regeneration (Zhang et al., 2014; Yanai et al., 2019). A case study by Mesimäki et al. demonstrated that autologous transplanted ASCs with BMP2 and beta-tricalcium phosphate reconstituted the defect area of the maxillary (Mesimäki et al., 2009). A similar study conducted by Wolff et al. and others showed successful reconstitution of the mandibular defects using tissue engineered constructs of autologous ASCs, beta-tricalcium phosphate (β-TCP) granules, recombinant human BMP-2 in a cohort of three patients (Wolff et al., 2013).

In addition to ASCs therapeutic potential, studies have shown that ASCs-Exo can stimulate bone regeneration through a paracrine mechanism (Zhu et al., 2021). Furthermore, in mouse models, decellularized adipose tissue hydrogels have shown promise, especially when combined with ASCs. The decellularized adipose tissue scaffolds promote bone regeneration and a higher volume of partially mineralized tissue and higher levels of collagen and osteopontin when the scaffolds are treated with either ASCs or osteogenic lineage induced ASCs and hydroxyapatite (Mohiuddin O. A. et al., 2019).

### Cartilage Regeneration

In general, cartilage has a narrow potential for regeneration (Tiku and Sabaawy, 2015). Of the different types of cartilage, articular cartilage is the most common therapy target, with joint injury and osteoarthritis (OA) being the main targets of regenerative medicine (Hulme et al., 2021). Clinical trials using autologous and allogeneic ASCs injections, ASCs-Exo, ASCs-CM to treat knee osteoarthritis have been reported to be well-tolerated and yield positive results in pain amelioration, decreased stiffness, and increased physical function with no severe side effects (Chen et al., 2021). A study conducted by Spasovski et al. showed that treating patients with autologous ASCs enhances their clinical scores and reduces pain levels (Spasovski et al., 2018). Another study by Pers and others demonstrated that patients who received autologous ASCs injections in the knee showed improvement due to the paracrine factors released from the cells inducing an anti-inflammatory response (Pers et al., 2018). A recent study conducted by Li and others described the use of ECM/SVF-gel fraction in cartilage defect repair as ASCs migrate from the gel and differentiate in a natural microenvironment, thus increasing the therapeutic potential of ASCs (Li et al., 2020). Furthermore, engineered cartilage is another area that is gaining interest, as ASCs and Poly ε-Caprolactone Scaffolds have successfully been used to regenerate cartilage *in vitro* for later transplantation into a mouse model (Nguyen and Vu, 2021).

### Spinal Cord Injuries (SCIs)

While most reports on the use of ASCs to treat spinal cord injuries have been performed in animal models, human clinical trials are underway. Takahashi et al. investigated the outcomes of treating SCIs with either BM-MSCs or ASCs in a murine model (Takahashi et al., 2018). Their data revealed comparable levels of motor function improvement in moderate SCI models. They also observed a higher survival rate after transplantation in the severe SCIs model and improved neuronal and vascular protection in the ASC-treated group (Takahashi et al., 2018). Another group explored using hASCs combined with low-level laser in neuropathic pain in experimental SCI models in rats. Their results demonstrated that combining low-level laser treatment with ASCs improved the level of the motor function recovery and had superior outcomes in alleviating SCI-induced allodynia and hyperalgesia; compared to groups treated with ASCs alone (Sarveazad et al., 2019). The therapeutic potential of mesenchymal stem cells isolated from either bone marrow, adipose tissue, or dental pulp was explored by another group in both a small (rats) and a large (pigs) animal model in spinal cord injury during the spinal contusion subacute period (Mukhamedshina et al., 2019). Applying ASCs in combination with fibrin matrix in the rat model provided significantly higher post-traumatic regeneration results than other MSCs. However, while the application of ASCs embedded in the fibrin matrix at the SCI site in pigs restored neural tissue integrity, no significant functional improvements were noted. A few case studies utilizing ASCs in human spinal cord injury patients have positive outcomes. The first human trial was performed in South Korea in 14 patients. It employed intrathecal transplantation of autologous ASCs with an 8-months follow-up. While the injury site and degree of impairments were diverse, none of the patients developed severe adverse effects from the transplantation procedure. While there were no differences in the areas of spinal damage as observed in the MRIs before and after the procedure, five patients showed an improved ASIA motor score, ten patients showed improved ASIA sensory score, and two patients that had no control over anal sphincters recovered it after 1- and 4-months post-treatment, respectively (Hur et al., 2016). Finally, the first report from a patient from the CELLTOP study, an ongoing multidisciplinary phase 1 clinical trial conducted at Mayo Clinic, has been released (Bydon et al., 2020). A 53-year-old male patient underwent an autologous intrathecal injection 11 months after a surfing injury., there was a progressive improvement in motor and sensory ASIA scores and the overall quality of life. There were no reports of adverse events within the 18-months follow-up period. Overall, these results highlight that, in general, the potential benefits of using ASCs for spinal cord injury.

### Stem Cell Therapy and COVID-19

ASCs are known for their potent immunomodulatory potential, which permits the cells to sense their microenvironment and respond according to the circumstances. Additionally, mesenchymal stem cells from diverse sources have proven effective in impairing viral replication and reducing the viral load (Rogers et al., 2020). Based on that, researchers are currently investigating the possibility of utilizing ASCs to treat COVID-19 infected patients. The primary complications from COVID-19 infection include damage to the lung tissue, excessive inflammation, and progression of lung fibrosis. Shi and others summarized early clinical testing of ASC injections, which highlights the ability of MSCs to reduce infiltration by immune cells and help repair the damaged tissue (Shi et al., 2021). A study conducted by Leng et al. showed that intravenous injection of stem cells was very effective in treating COVID-19 patients with pneumonia by decreasing pro-inflammatory cytokines and increasing the anti-inflammatory cytokines such as IL-10, thus promoting lung repair (Leng et al., 2020). A study by Gentile and Sterodimas investigated the use of autologous or allogeneic ASCs to treat severe COVID-19 cases (Gentile and Sterodimas, 2020). ASC infusions were shown to inhibit the over-activation of the immune system, promoting endogenous repair by improving the lung microenvironment. Although ASCs have shown promising outcomes, the type of stem cells, the dose administered, the interval of time, and the delivery mechanism should be optimized for future clinical applications. Some of these issues may be addressed as results of ongoing COVID-19 clinical trials are published.

## Future Applications for ASCs

### Nanotherapeutics: 3D Scaffolds With ASCs

Nanotechnology has opened a new opportunities for novel applications of stem cells for numerous diseases, such as cardiovascular, neurological, vascular diseases, diabetes and inflammation (Masoudi Asil et al., 2020; Dong et al., 2021; Sarathkumar et al., 2021). It is worth noting that NPs are naturally occurring products from ASCs and are secreted as EVs (Wang et al., 2009; Dong et al., 2021). ASC-derived NPs offer novel, non-invasive methods and provide much information about tissue repair and develop a precise method for targeted therapy (Qiu et al., 2018; Dong et al., 2021). Beyond that combining stem cells, with scaffolds is driving the creation of organoids, organ-on-a-chip systems and lab grown organs and tissues.

Scaffolds, synthetic or natural “biologically-derived”, to be used in tissue engineering applications, must recapitulate the extracellular matrix (ECM) and imitate an *in vivo* like microenvironment favorable for stem cell attachment and proliferation. Synthetic scaffolds are made up of polyesters, polyethers, polyethylene glycol and polylactic acid (PLLA) (Gibler et al., 2021b; Reddy et al., 2021), while natural scaffolds “commonly used with ASCs” are comprised of collagen, fibrin, gelatin, vitronectin, laminin, alginate, hyaluronic acid, or decellularized materials (DAT) (Sadeghi-Ataabadi et al., 2017; Vinson et al., 2017; Kook et al., 2018; Mohiuddin O. A. et al., 2019; Colle et al., 2020; O’Donnell et al., 2020). The central characteristics of the scaffolds include the composition and porosity necessary to promote cell adhesion, proliferation and differentiation; fibrosity and stiffness; biocompatibility with the tissue and biodegradability with a negligible amount of toxicity or inflammation *in vivo* (Dhandayuthapani et al., 2011; Reddy et al., 2021). Several studies have incorporated ASCs into 3D scaffolds and demonstrated their ability to adhere, migrate and differentiate into specific cell lineages. The choice of nanomaterial used in generating ASC scaffolds should be carefully considered as it might influence the differentiation ability of stem cells. In addition to the ECM-ASC interactions in a scaffold, growth factors (such as VEGF, bFGF and TGF-β) can effectively be integrated to enhance the therapeutic potential by enhancing the proliferation and differentiation of stem cells (Howard et al., 2008; Sell et al., 2011; Sadeghi-Ataabadi et al., 2017). Similarly, studies have demonstrated the function of ASCs seeded on PRP fibrin enriched scaffolds in cartilage repair and tendon regeneration (Drengk et al., 2009; Barbon et al., 2019), as well as skin graft transplantation by inducing tissue angiogenesis (Wang et al., 2019; Gao et al., 2020). Nair and others showed that MSCs incorporated into graphene oxide (GO) scaffolds enhanced osteogenic differentiation. Thus, they may be used for bone regeneration in orthopedic applications (Nair et al., 2015). A similar test of GO scaffolds demonstrated their ability to induce neuronal differentiation of mesenchymal stem cells (Kim et al., 2015). In another study, PLLA and poly 3-hydroxybutyrate scaffolds combined with dental pulp-derived MSCs are a potential treatment of cardiovascular diseases (Castellano et al., 2014). Yin and others demonstrated ASC differentiation into chondrocytes in the context of PLGA gelatin scaffolds supplemented with TGF-β1 (Yin et al., 2015).

Decellularized adipose tissue hydrogels (hDAT) impregnated with stem cells have been widely tested as treatments for spinal cord and peripheral nerve injury, wound healing, myocardial infarction, cartilage repair, and bone tissue engineering. Mohiuddin and others have shown that DAT hydrogels support ASC proliferation and multilineage differentiation capabilities. Additionally, the ASCs remodeled the microstructure of the hydrogels, making them more compatible for *in vivo*-like adipose tissue regeneration (Mohiuddin O. A. et al., 2019). The use of decellularized scaffolds and ASCs in regenerative medicine is promising. However, more research into the method of preparation, “decellularization and sterilization,” should be optimized for each use, as it will affect the quality of stem cells attachment and differentiation. Yang et al. described the different decellularized and sterilization techniques used in the preparation of DAT hydrogels as well as the problems that needs to be resolved before their application in clinicals trials (Yang et al., 2020). The decellularization methods involve a combination of biological (enzymatic digestion DNase/RNase) chemical (isopropanol, Triton X and sodium chloride), and physical (freeze and thaw cycles, homogenization) treatments. The sterilization techniques mainly included 70% ethanol, penicillin and streptomycin, ethylene oxide and UV light (Song et al., 2018; Thomas-Porch et al., 2018; Yang et al., 2020). Although these methods have been proven to be effective in decellularization, the use of certain detergents and enzymes cause the loss of the main ECM components such as collagen, laminin and glycosaminoglycans (GAGs) and destruction of protein-protein interactions. Yang et al. also mentioned that residues of chemical and enzymatic substances or cell debris might affect stem cell adhesion differentiation of stem cells as well as induce an immune response. Thus, optimizing protocols for adipose tissue decellularization will be important to develop 3D biomaterial scaffolds that serve as effective *in vitro* models of the pathophysiology of various diseases and can be used to screen novel therapeutics and reduce the use of experimental animal models.

### Microphysiological Systems: “Organ-on-a-Chip” Technology

Microphysiological systems (MPS) are an advanced technology that permits researchers to study cell-cell, cell-ECM and cell-tissue interactions in an in the vivo-like dynamic microenvironment. MPS uses either 3D organoids “assembled from cell-cell interactions in a scaffold-free manner” or on scaffolds to develop “organ-on-a-chip” systems applicable for drug screening and assessment of novel treatments (Zhang et al., 2017; Ronaldson-Bouchard and Vunjak-Novakovic, 2018; Wu et al., 2020; Marrazzo et al., 2021). In MPS, stem cells can be seeded in layers or interconnected cell culture chambers linked by microchannels filled with media (particular to the cell type). They can also incorporate mechanical and biochemical stimuli to replicate human tissues’ physiology and biology *in vivo*. The microchannels included in MPS are designed to control the flow of the media between the chambers, and they can be sealed or perfused depending on the type of planned experiments. Although MPS has been widely investigated, only a few published articles presently report using stem cells, including ASCs (Zhang et al., 2017; Paek et al., 2019; O’Donnell et al., 2020; Marrazzo et al., 2021). Kefallinou and others demonstrated the generation of bone marrow-on-a-chip (BMoC), which may be useful as a system to study the stromal niche in diseases such as SLE (Kefallinoua et al., 2020). Another article published by Lavrentieva described a microfluidic gradient system using hASCs and human umbilical cord vein endothelial cells (HUVECs) were embedded in a methacrylated gelatin (GelMA) hydrogel to study the morphological changes and cellular interaction in a stem cell niche and how the stiffness of the gels plays a significant role in developing scaffolds used for clinical applications (Lavrentieva et al., 2020). An MPS combining human ASCs and human adipose microvascular endothelial cells (hAMECs) in a collagen and fibrin hydrogel scaffold to create a 3D microvascular-like network that recapitulates the interaction between the cell types and facilitates the study of vascular inflammation in a dynamic fluid system has been reported (Paek et al., 2019). The data collected using the MPS will provide novel research tools for developing preclinical models representing the physiological niche and potential treatment of vascular and metabolic diseases. However, more research is needed on how mechanical stimulus affects stem cell niche development under physiological conditions. One study has shown that interstitial shear stress in a fluidic system might decrease the ASCs’ ability to undergo adipogenesis (Bender et al., 2020).

### Challenges and Limitations

Human pre-clinical and clinical trials are underway to provide treatment for multiple diseases, including chronic conditions such as systemic sclerosis, neurological degenerative diseases, non-healing wounds as in diabetic ulcers, or the treatment of fistulas in patients with Chron’s Disease, neurodegenerative diseases, atherothrombotic diseases, chronic kidney disease, degenerative osteoarthritis, and enhance recovery after ligament and tendon injuries, and promote the field of reconstructive plastic surgery in breast reconstruction and skin rejuvenation (Shukla et al., 2020). Nevertheless, there are still many hoops to jump ahead and a long way to get these therapies to move from clinical trials to FDA-approved treatments. One of the limitations for the usage of ASCs in regenerative medicine is the donor’s age, body mass index (BMI) and health conditions (underlying disease or comorbidities), which might result in reduced immunomodulatory abilities of main regulatory factors (Strong et al., 2013; Barwinska et al., 2018). Thus, ASCs should be fully characterizing and thoroughly screened for *in vitro* aging and inflammatory markers that might affect their regenerative abilities and hinder their usage in clinical application. Further, in-depth research is needed to study the effect of nanomaterial, pore size, stiffness, biodegradability on stem cell proliferation, migration and differentiation while developing scaffolds as a novel therapeutic tool that imitates the *in vivo* microenvironment. One of the limitations for using ASCs scaffolds in an MPS is their proliferation, migration and capability to multi-differentiate into particular lineages in a co-culture system for an extended period.

## Conclusion

ASCs have therapeutic potential in regenerative medicine and applications in tissue engineering. While the biologic properties of ASCs are not yet fully delineated, the cells are under clinical investigation in human trials for an array of diseases. ASCs have been widely studied for their immunomodulatory effects, anti-fibrotic, anti-apoptotic, and anti-oxidative capabilities in preclinical and human clinical trials. Additionally, the ASC secretome (conditioned media or exosomes) has demonstrated similar effectiveness in regenerative medicine applications. However, the field must be cognizant of the inherent donor-to-donor variation, which can significantly impact the therapeutic potential of ASCs and ASC-derived products regarding their growth and differentiation efficiency, which will directly impact their therapeutic potential as cell therapies their effectiveness in more complex 3D systems.

Furthermore, the emerging technology combining stem cells with scaffolds or microfluidic chip technologies provides novel opportunities to develop more sophisticated and effective treatments with minimal risks and side effects. Therefore, ASC-based scaffolds and “organ-on-a-chip” models represent an advanced technology in regenerative medicine that will provide researchers with new tools to treat numerous diseases that conventional medicine could not effectively cure. However, investigators must address several considerations as the more complex 3D systems develop including, cell types required for their creation, development of standardized methodologies for their generation, criteria for characterization of how effectively it recapitulates an intact organ, and readouts for biologic and efficacy assessments.
